# Enhancing the Catalytic Activity of Glycolate Oxidase from *Chlamydomonas reinhardtii* through Semi-Rational Design

**DOI:** 10.3390/microorganisms11071689

**Published:** 2023-06-28

**Authors:** Yingting Feng, Shuai Shao, Xueting Zhou, Wan Wei, Xun Liu, Yi Tang, Yuhao Hua, Jianyong Zheng, Yinjun Zhang, Xiangxian Ying

**Affiliations:** Key Laboratory of Bioorganic Synthesis of Zhejiang Province, College of Biotechnology and Bioengineering, Zhejiang University of Technology, Hangzhou 310014, China; fyt9191@163.com (Y.F.); ss1964241742@163.com (S.S.); sadie598851321@163.com (X.Z.); zjut2112105022@163.com (W.W.); lx0925124X@163.com (X.L.); ty12150102@163.com (Y.T.); hyhua0616@163.com (Y.H.); zjy821212@zjut.edu.cn (J.Z.)

**Keywords:** glycolate oxidase, *Chlamydomonas reinhardtii*, fusion enzyme, semi-rational design, methyl glyoxylate, enzymatic oxidation

## Abstract

Glycolate oxidase is a peroxisomal flavoprotein catalyzing the oxidation of glycolate to glyoxylate and plays crucial metabolic roles in green algae, plants, and animals. It could serve as a biocatalyst for enzymatic production of glyoxylate, a fine chemical with a wide variety of applications in perfumery, flavor, and the pharmaceutical and agrochemical industries. However, the low catalytic activity of native glycolate oxidase and low levels of active enzyme in heterologous expression limit its practical use in industrial biocatalysis. Herein, the glycolate oxidase from *Chlamydomonas reinhardtii* (CreGO) was selected through phylogenetic tree analysis, and its low level of soluble expression in *E. coli* BL21(DE3) was improved through the use of the glutathione thioltransferase (GST), the choice of the vector pET22b and the optimization of induction conditions. The semi-rational design of the fusion enzyme GST-Gly-Ser-Gly-CreGO led to the superior variant GST-Gly-Ser-Gly-CreGO-Y27S/V111G/V212R with the *k*_cat_/*K*_m_ value of 29.2 s^−1^·mM^−1^, which was six times higher than that of the wild type. In contrast to GST-Gly-Ser-Gly-CreGO, 5 mg/mL of crude enzyme GST-Gly-Ser-Gly-CreGO-Y27S/V111G/V212R together with 25 μg/mL of catalase catalyzed the oxidation of 300 mM of methyl glycolate for 8 h, increasing the yield from 50.4 to 93.5%.

## 1. Introduction

Glycolate oxidase (EC 1.1.3.15; GO) is an FMN-dependent enzyme catalyzing the oxidation of glycolate to glyoxylate at the expense of oxygen [1,2]. The FMN-mediated reaction catalyzed by GO consists of two stages: the reductive half-reaction, which leads to the oxidation of glycolate and the concomitant reduction in FMN; and the oxidative half-reaction wherein the flavin is oxidized by O_2_ to generate FMN and hydrogen peroxide [1,2,3]. Glycolate oxidase plays crucial roles in the metabolism of plants, animals, and green algae. Glycolate oxidase is involved in the photorespiratory cycle in green algae and plants, while the enzyme is responsible for the production of oxalate in animals. Besides its physiological roles, glycolate oxidase as a biocatalyst converts glycolate to glyoxylate, a fine chemical with diversified uses [4,5,6,7]. As the starting material, glyoxylate together with isobutanol was used for the synthesis of (*R*)-pantolactone through the combination of an asymmetric organocatalytic aldol reaction with subsequent biotransformation [7]. In contrast to glycolic acid, the oxidation of methyl glycolate offered the advantages of less pH change, easy product isolation, and direct process integration for subsequent organic synthesis [8]. Since methyl glycolate is not a natural substrate, glycolate oxidases in nature often have the problems of low catalytic activity and poor stability. Thus, expanding the glycolate oxidase sources is essential to meet the requirements of industrial biocatalysis on activity [9,10,11,12].

Aiming for industrial application, it is necessary to achieve the cost-effective production of glycolate oxidase through high-level heterologous expression [13,14,15]. Many expression systems are available for recombinant protein production, and *Escherichia coli* is often the first choice host because of the rapid growth and high yields of recombinant protein [15,16]. As a peroxisomal flavoenzyme, glycolate oxidase is encoded in the nucleus, synthesized in the cytoplasm and delivered to peroxisome [17]. The heterologous expression of glycolate oxidase in *E. coli* often results in the accumulation of insoluble aggregates [18,19,20]. Various factors have been explored to improve the soluble expression of recombinant protein in *E. coli*, including the choice of vector and host strain, the optimization of culturing conditions, and the use of fusion tags and chaperones [13,15,21,22]. For instance, glutathione S-transferase (GST) is widely used as a solubility-enhancing tag as well as an affinity tag [22]. The vector alteration to pET22b resulted in the production of spinach glycolate oxidase in the periplasm [19]. Low-temperature induction usually mitigates the formation of insoluble aggregates [23]. It would achieve superior soluble expression of recombinant proteins through the combination of different strategies.

Directed evolution has been proven to be an effective approach to activity enhancement [8]. The directed evolution of spinach glycolate oxidase was performed to generate the mutant library by error-prone PCR and the mutant screening was judged on the evolved H_2_O_2_ accompanied by alcohol oxidation. The catalytic efficiency of the double substitution M267T/S362G was nearly two times higher than that of the wild type. In contrast to the directed evolution, the knowledge-based semi-rational design could significantly improve the efficiency of enzyme tailoring. Combining with computational predictive algorithms, the “smart” library design utilizes structural and functional knowledge to focus on promising target sites and limits amino acid diversity for protein engineering [24,25,26,27,28]. In recent decades, structural information on glycolate oxidase has accumulated and various computational tools have emerged [1,2,25,29,30,31], offering solid feasibility of semi-rational design.

**Scheme 1:**
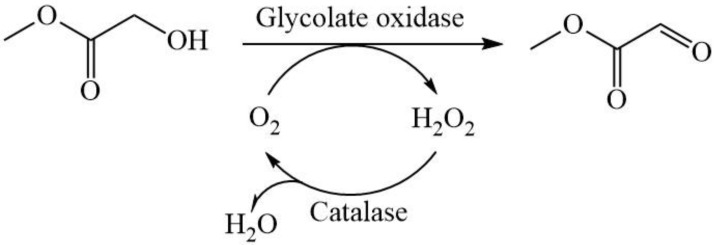
The oxidation of methyl glycolate to methyl glyoxylate through the catalysis of glycolate oxidase and catalase.

Through phylogenetic analysis, the glycolate oxidase from *Chlamydomonas reinhardtii* (CreGO) was selected and then over-expressed in *E. coli*. The efforts including the use of the fusion tag glutathione thioltransferase (GST), the choice of pET22b, and low-temperature induction improved the soluble expression of CreGO. Furthermore, the semi-rational design of CreGO focused on the key residues in the substrate-binding pocket and effectively enhanced both the catalytic activity and enzyme productivity. Finally, the resulting variant with triple substitution demonstrated superior catalytic performance in the oxidation of 300 mM methyl glycolate to methyl glyoxylate (Scheme 1).

## 2. Materials and Methods

### 2.1. Chemicals, Genes, Plasmids, and Strain

The chemicals of analytical grade were purchased from Sigma-Aldrich (Shanghai, China). The ClonExpress MultiS One-Step Cloning Kit was bought from Vazyme Biotech Co., Ltd. (Nanjing, China). The codon-optimized genes encoding the glycolate oxidase CreGO (GenBank accession No.: BAK61668.1), GST from *Pseudomonas* sp. Ag1 (WP_008430144.1) and catalase from *Acinetobacter* sp. YS0810 (AHY00946.1) were synthesized by Tsingke Biotechnology Co., Ltd. (Hangzhou, China). The expression vectors pET28a and pET22b were used for over-expression. The strain *E. coli* BL21 (DE3) was used as the host for cloning and over-expression, respectively.

### 2.2. Expression of CreGO and GST

The gene encoding CreGO was inserted into the sites *Eco*R I/*Hin*d III of the vector pET28a, and the resulting plasmid was designated as pET28a-*CreGO*. The recombinant plasmid pET28a-*CreGO* was transformed into the host strain *E. coli* BL21(DE3), giving the recombinant strain *E. coli* BL21(DE3)/pET28a-*CreGO*. Similarly, the gene encoding GST was inserted into the sites *Eco*R I/*Hin*d III of the vector pET22b, and the resulting plasmid was designated as pET22b-*GST*. The recombinant plasmid pET22b-*GST* was transformed into the host strain *E. coli* BL21(DE3), giving the recombinant strain *E. coli* BL21(DE3)/pET22b-*GST*.

An LB medium containing 50 µg/mL of antibiotics was routinely used for culturing recombinant *E. coli* strains at 37 °C until the OD_600_ of 0.8. Specifically, the strains containing the vector pET22b or pET28 were treated with 50 µg/mL of ampicillin or kanamycin, respectively. After the optimization, the induction was initiated by the addition of 0.1 mM of isopropyl β-D-thiogalactoside (IPTG) and then the strains were cultured at 20 °C for 15 h. At the end of incubation, the cells were washed twice using Tris-HCl buffer (50 mM, pH 8.0), harvested by 8000× *g* centrifugation at 4 °C for 10 min and stored at −20 °C for further use. To visualize the expression of CreGO and GST, 12% (*w*/*v*) sodium dodecyl sulfate-polyacrylamide gel electrophoresis (SDS-PAGE) was used [32].

### 2.3. Fusion Expression of CreGO and GST

The fusion genes encoding CreGO and GST were assembled by multiple overlap extension PCR [8]. Using the pair of primers pET22b-GST-F and pET22b-GST-GSG-R (Table S1), the linker Gly-Ser-Gly (GSG) was introduced downstream of GST. Similarly, the gene encoding CreGO was flanked by the linker GSG together with the overlap region to GST and the overlap region to pET22b using the pair of primers GSG-CreGO-F and CreGO-R (Table S1). The plasmids pET22b-*GST* and pET28a-*CreGO* served as the corresponding template. The PCR program was composed of the following steps: 98 °C for 5 min, 30 cycles of 98 °C (10 s), 58 °C (10 s) and 72 °C (30 s), and a final 5 min extension at 72 °C. The PCR products were digested by *Dpn* I to remove the template and then purified. Through Exnase-II-derived homologous recombination in the ClonExpress MultiS One Step Cloning Kit, the gene encoding CreGO and the linear pET22b-*GST-GSG* were ligated to form the recombinant plasmid pET22b-*GST-GSG-CreGO*. The recombinant plasmid was transformed into the host strain *E. coli* BL21(DE3), and the resulting strain was grown in LB medium containing 50 µg/mL ampicillin. After the induction of the fusion enzyme, the expression of the fusion enzyme was visualized through SDS-PAGE analyses.

### 2.4. Homology Modeling and Molecular Docking

The structural model of CreGO was built on the AI-aided web server iDrug (https://drug.ai.tencent.com, accessed on 25 April 2021), using the crystal structure of the glycolate oxidase from spinach (PDB ID: 1GOX) as a template [33]. The model was evaluated by the software SAVES v6.0 (http://saves.mbi.ucla.edu/, accessed on 2 May 2021). The protein model was protonated and energy minimized using Schrödinger’s protein preparation function to output the optimal model; similarly, for small molecules, they were optimized using the ligprep after being drawn on ChemDraw3D 8.0 software. FMN and methyl glycolate as a ligand were docked with CreGO using the AutoDock Vina 1.2.3 program (The Scripps Research Institute, La Jolla, CA, USA) [34]. The optimal configuration and resulting substrate-enzyme complex were further processed using PyMOL 2.0 software (The PyMOL Molecular Graphics System, Schrödinger, LLC., New York, NY, USA).

### 2.5. Site-Directed/Saturation Mutagenesis

Following the whole-plasmid mutagenesis protocol, site-directed/saturation mutagenesis of GST-GSG-CreGO was run using the recombinant plasmid pET22b-*GST*-*GSG*-*CreGO* as a template. The primers for site-directed/saturation mutagenesis are listed in Tables S2–S5. The PCR conditions were as follows: 98 °C for 5 min, and then 30 cycles (98 °C for 15 s, 58 °C for 15 s, 72 °C for 1 min), 72 °C extension for 5 min. In the first round of iterative saturation mutagenesis, the recombinant plasmid pET22b-*GST*-*GSG*-*CreGO*-*Y27S* was used as a template; the second round of iterative saturation mutations was performed on the basis of pET22b-*GST*-*GSG*-*CreGO*-*Y27S/V212R*. The PCR products were digested by *Dpn* I at 37 °C for 1 h and then directly transformed into *E. coli* BL21(DE3) competent cells to generate the variants.

### 2.6. Purification of GST-GSG-CreGO and Its Variants

The cells expressing GST-GSG-CreGO and its variants were re-suspended in 100 mM PBS buffer containing 5 mM EDTA and 200 g/L sucrose (pH 6.5), incubated at 20 °C for 30 min and then disrupted by sonication for 20 min. After centrifugation, the cell debris pellets were discarded, and the resulting supernatant was used as a crude enzyme. The cell-free extracts were applied for Ni-NTA chelating affinity chromatography. Unbound proteins were washed off by applying the binding buffer (5 mM imidazole and 300 mM NaCl dissolved in 50 mM Tris-HCl, pH 8.0). The enzyme GST-GSG-CreGO or its variant was eluted out by applying the elution buffer (250 mM imidazole and 300 mM NaCl dissolved in 50 mM Tris-HCl, pH 8.0). The pH value of the buffer containing imidazole was measured after imidazole dissolution. The purity of the purified enzyme was verified using SDS-PAGE as described previously. The purified GST-GSG-CreGO and its variants were desalted with 50 mM Tris-HCl buffer (pH 8.0) and stored at −20 °C for further investigation.

### 2.7. Activity Assay and Determination of Kinetic Parameters

The product glyoxylate from the oxidation of glycolate reacts with phenylhydrazine hydrochloride to form glyoxy phenylhyarazone, which has strong absorbance at 324 nm. Thus, the glycolate oxidase activity was measured at 40 °C by monitoring changes in absorbance at 324 nm. All enzyme assays were performed in triplicate. The assay mixture (1 mL) consisted of 50 μg crude enzyme or 1 μg purified enzyme, 10 mM phenylhydrazine hydrochloride, 10 mM sodium glycolate and 100 mM PBS buffer (pH 6.5). The enzyme assays began with the addition of sodium glycolate. The unit of glycolate oxidase activity is defined as the amount of enzyme required to convert 1 μmol sodium glycolate to sodium glyoxylate per min at 40 °C and pH 6.5. The bicinchoninic acid (BCA) method was used to determine the protein concentration and bovine serum albumin was used as the standard protein [35]. Kinetic parameters of the purified CreGO and its variant were determined within the substrate concentration range from 0 to 100 mM. According to Michaelis–Menton kinetics, the parameters *K*_m_ and *V*_max_ were calculated through the curve fitting using Origin Pro software (Version 8.5).

### 2.8. Expression of Catalase and Its Activity Determination

The gene encoding catalase from *Acinetobacter* sp. YS0810 was inserted into the sites *Eco*R I/*Hin*d III of the vector pET28a, and the resulting plasmid was designated as pET28a-*CAT*. The recombinant plasmid pET28a-*CAT* was transformed into the host strain *E. coli* BL21(DE3), giving the recombinant strain *E. coli* BL21(DE3)/pET28a-*CAT*. Following the procedures in Section 2.2 and Section 2.6, the recombinant strain *E. coli* BL21(DE3)/pET28a-*CAT* was induced and its crude enzyme was prepared.

As the byproduct from the oxidation of methyl glycolate, H_2_O_2_ underwent absorbance at 240 nm. The crude recombinant catalase was used to decompose H_2_O_2_. The catalase activity was measured at 40 °C and pH 6.5 by monitoring changes in absorbance at 240 nm. The assay mixture (1 mL) consisted of 10 mM H_2_O_2_, 0.25 μg crude enzyme and 100 mM PBS buffer (pH 6.5). The unit of the catalase activity was defined as the amount of enzyme required to degrade 1 μmol H_2_O_2_ per min at 40 °C and pH 6.5.

### 2.9. Oxidation of Methyl Glycolate to Methyl Glyoxylate

The reaction mixture (15 mL) consisted of 300 mM methyl glycolate, 5 mg/mL crude glycolate oxidase, 25 μg/mL crude catalase and 100 mM PBS buffer (pH 6.5). The reaction was run in triplicate at 40 °C and 1000 rpm for 8 h, while the oxygen aeration was set as 1.35 L/h. When the reaction was terminated, the substrate and product in the reaction mixture were determined by HPLC analyses.

### 2.10. HPLC Analyses

The sample pretreatment to remove the proteins was required for reliable HPLC analyses. To denature the proteins, a 200 μL reaction mixture was mixed with 1800 μL of methanol. The diluted samples were centrifuged at 10,000× *g* for 5 min and the protein precipitate was discarded. The resulting supernatant was filtered through a 0.22 µm filter. The filtrate was collected and further applied to 10 kDa ultrafiltration driven by the centrifugation at 5000× *g* for 20 min. The filtrate after ultrafiltration was subjected to HPLC analyses.

The HPLC instrument (Waters 1525, Waters Corporation, Milford, MA, USA) equipped with a UV detector (Waters 2489, Waters Corporation, Milford, MA, USA) and Welchrom C18 (250 mm × 4.6 mm, 5 μm, Welch Materials, Inc., Shanghai, China) was used for the determination of methyl glycolate and methyl glyoxylate. The mobile phase (1 L) consisted of 100 mL acetonitrile and 900 mL phosphate buffer (50 mM, pH 6.0) at a flow rate of 0.6 mL/min. The detection was set as 210 nm at 30 °C, and the injection volume was 10 μL. The retention times for methyl glycolate and methyl glyoxylate were 4.07min and 2.51 min, respectively (Figure S1).

## 3. Results and Discussion

### 3.1. Selection of Glycolate Oxidase

**Figure 1:**
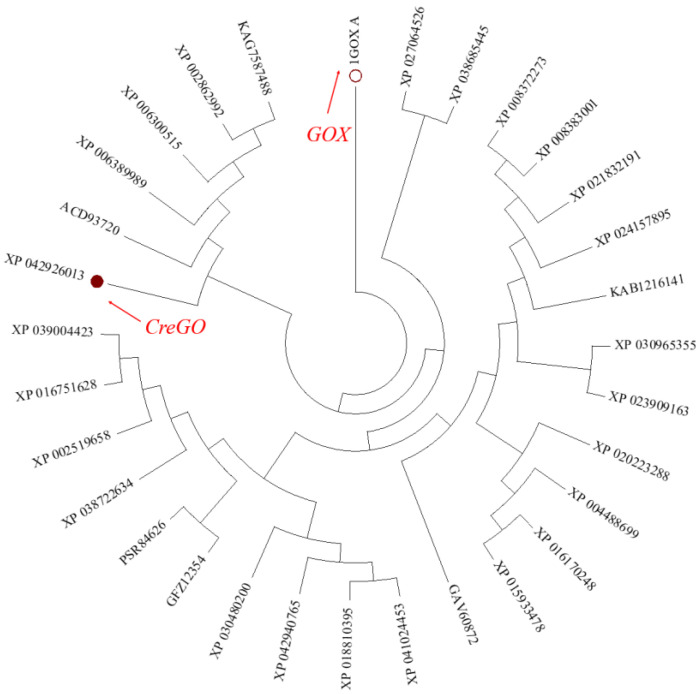
Phylogenetic tree of glycolate oxidase genes in plants and green algae. On the basis of amino acid sequences searched from the NCBI database, a phylogenetic tree was constructed with the neighbor-joining methodology using the software MEGA 7.0.

During the past forty years, spinach glycolate oxidase GOX (PDB No.: 1GOX) has been extensively studied, accumulating plenty of structural and functional information [8,9,18,19,29,30]. However, its catalytic activity on the oxidation of methyl glycolate was relatively low when GOX was used as a biocatalyst [8]. Thus, it is necessary to expand the glycolate oxidase sources to meet the requirements of industrial biocatalysis. Using the amino acid sequence of GOX as a probe, 30 potential glycolate oxidases with 50~99% identity were searched from the NCBI database and subjected to phylogenetic tree construction (Figure 1). Among them, the glycolate oxidase from *Chlamydomonas reinhardtii* (CreGO) was unique, sharing a similarity of 69% and 50% of its identity with the amino acid sequence of GOX [11]. As unicellular green algae, *Chlamydomonas reinhardtii* is evolutionarily closer to *E. coli* than spinach [36]. Although the enzymatic properties of CreGO have not been investigated [11], it is believed that achieving soluble expression of GreGO in *E. coli* would be easier than that of GOX. Thus, the enzyme CreGO was selected for the subsequent study.

### 3.2. Improved Soluble Expression of CreGO

**Figure 2:**
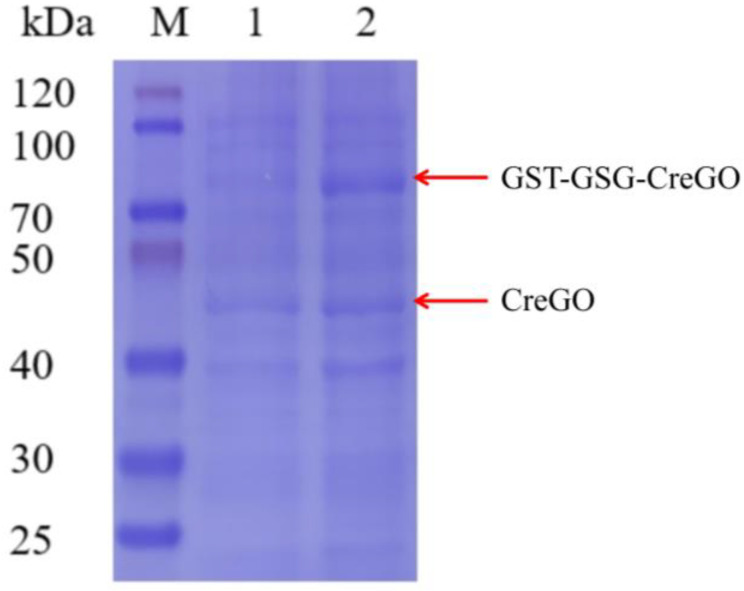
SDS-PAGE analyses of crude enzymes CreGO and GST-GSG-CreGO. Lane M, standard molecular mass proteins; lane 1, CreGO; lane 2, GST-GSG-CreGO. The proteins were visualized by staining with Coomassie brilliant blue R-250.

**Figure 3:**
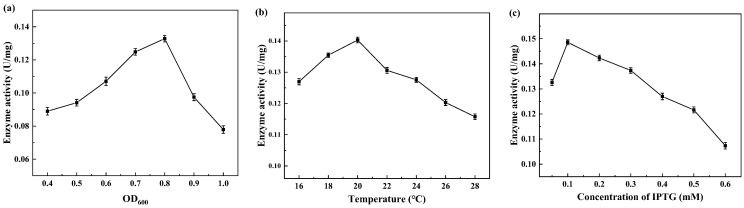
Factors affecting the induction of the strain *E. coli* BL21 (DE3)/PET22b-*GST*-*GSG*-*CreGO*. (**a**) The cell density to initiate the induction; (**b**) temperature; (**c**) the concentration of IPTG. Standard deviations are indicated in the diagram (*n* = 3).

The expression of CreGO was initially conducted through the construction of the recombinant strain *E. coli* BL21(DE3)/pET28a-*CreGO*. After induction and cell disruption, the crude enzyme activity was determined to be 0.06 U/mg. The use of GST as the fusion tag and the choice of pET22b proved able to facilitate the soluble expression [13,19,22]. The fusion enzyme GST-GSG-CreGO was constructed using the linker GSG, and its active expression was achieved through the construction of the strain *E. coli* BL21 (DE3)/pET22b-*GST*-*GSG*-*CreGO*. The SDS-PAGE analysis indicated that the size of CreGO and GST-GSG-CreGO were consistent with the calculated values (46.4 kDa and 72.3 kDa), respectively (Figure 2). The crude enzyme activity from the strain *E. coli* BL21 (DE3)/pET22b-*GST*-*GSG*-*CreGO* was increased up to 0.13 U/mg, demonstrating improved soluble expression. To further enhance the expression level of *GST*-*GSG*-*CreGO*, the induction conditions of the strain *E. coli* BL21 (DE3)/pET22b-*GST*-*GSG*-*CreGO* were investigated, including the cell density to initiate the induction, temperature, and concentration of IPTG. When the cell density OD_600_ to initiate the induction was tested from 0.4 to 1.0, the highest crude enzyme activity (0.14 U/mg) was observed at the OD_600_ of 0.8 (Figure 3a). The effect of temperature on induction was tested within the range of 16~28 °C, and the best expression level was observed at 20 °C (Figure 3b). In addition, the concentration of IPTG was optimized to be 0.1 mM (Figure 3c). Under the optimized induction conditions, the crude enzyme activity reached up to 0.15 U/mg, suggesting the effectiveness of combined strategies.

### 3.3. Homologous Modeling and Alanine Scanning of CreGO

**Figure 4:**
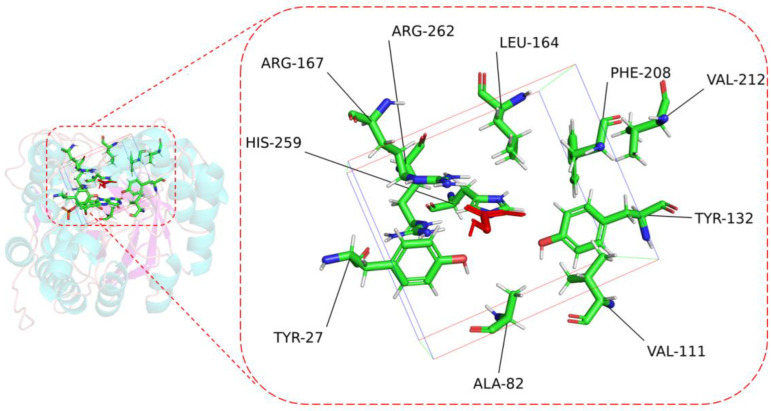
The key residues of CreGO within 4 Å vicinity when the ligand was taken as the center. Red, methyl glycolate. The frame represents 4 Å vicinity when the ligand was set as the center.

**Figure 5:**
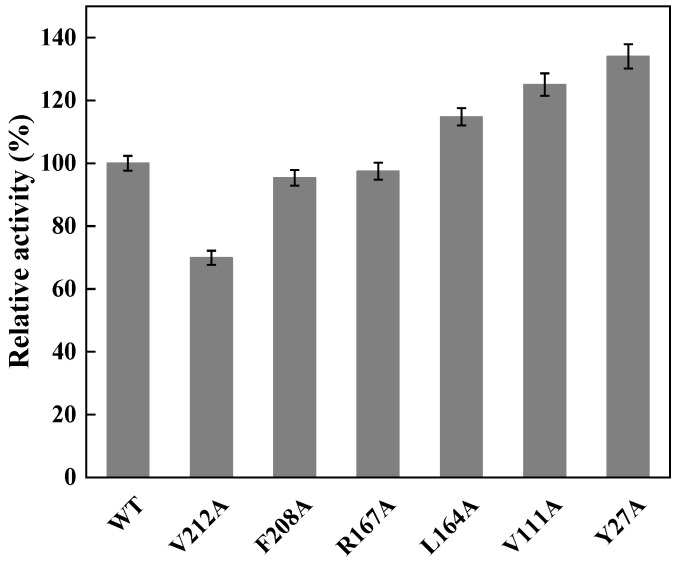
Relative activities of crude CreGO and its variants in alanine scanning. WT represents the fusion enzyme GST-GSG-CreGO. The relative activity of 100% represents 0.15 U/mg. Standard deviations are indicated in the diagram (*n* = 3).

To perform the semi-rational design of CreGO in the fusion enzyme GST-GSG-CreGO, the structure model of CreGO was built through the AI-aided web server iDrug (Figure S2). In the model evaluation, the Ramachandran plot analysis indicated that the CreGO model was reliable (Figure S3). To identify the key residues on activity, molecular docking of CreGO and the ligand methyl glycolate was performed using the program Autodock Vina (Figure 4). Compared with the crystal structure of 1GOX [37], the active sites of CreGO were identified to be Y132, H259 and R262. Taking the docked ligand as the center, the residues within a 4 Å vicinity (Y27, A82, V111, Y132, L164, R167, F208, V212, H259 and R262) were considered as putative hot spots (Figure 4) [38]. Except for the active sites (Y132, H259 and R262) and A82, the putative hot spots (Y27, A82, V111, L164, R167, F208 and V212) were subjected to alanine scanning [39]. The expression level of six variants from alanine scanning was similar to that of the wild type (Figure S4). The evaluation of the variants was based on the determination of the crude enzyme activity using sodium glycolate as the substrate. The crude enzyme activities of R167A and F208A were close to that of the wild type (Figure 5). Compared with the wild type, the relative activities of L164A, V111A and Y27A were 114.7%, 125.1% and 134.0%, respectively. In addition, the crude enzyme activity of V212A was only 69.9% that of the wild type, suggesting that it also played a pivotal role in the activity. Thus, the key residues Y27, V111 and V212 were chosen for subsequent site-saturation mutagenesis and iterative saturation mutation.

### 3.4. Site-Saturation Mutagenesis of the Residue Y27 and Iterative Saturation Mutagenesis of the Residues V111 and V212

**Figure 6:**
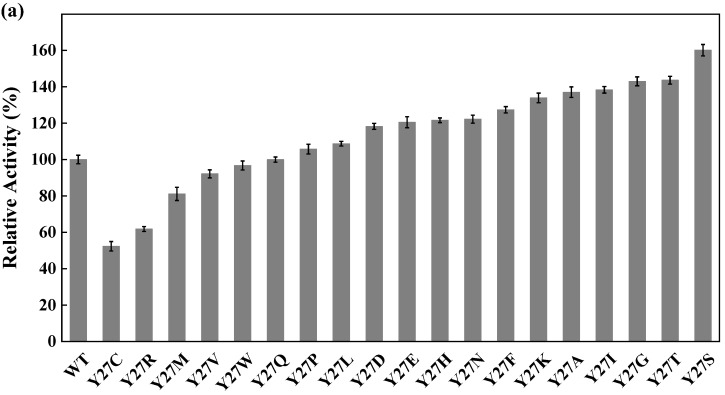

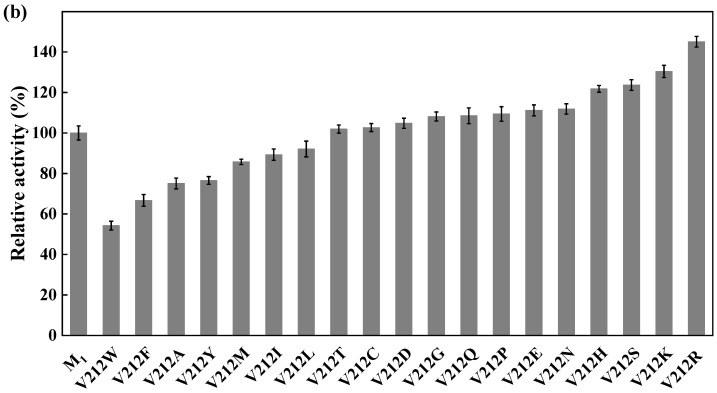

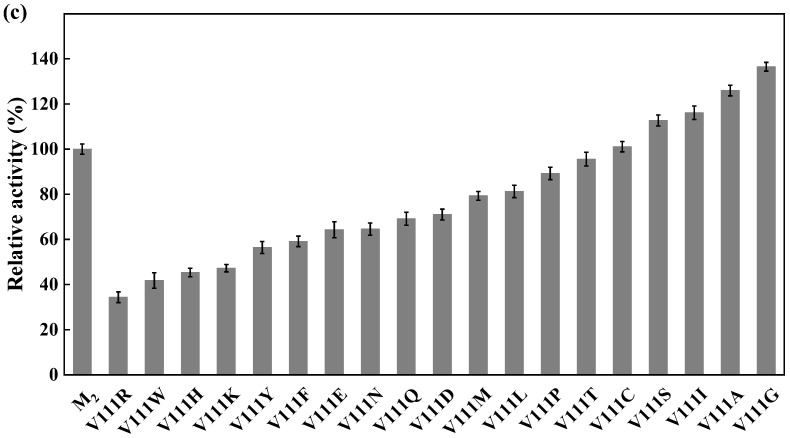
Iterative saturation mutation of the residues Y27 (**a**), V212 (**b**) and V111 (**c**). WT, GST-GSG-CreGO; M_1_, GST-GSG-CreGO-Y27S; M_2_, GST-GSG-CreGO-Y27S/V212R. Standard deviations are indicated in the diagram (*n* = 3).

Site-directed saturation mutagenesis was performed to determine the best substitution for the residue Y27. The wild type and the corresponding 19 variants were expressed in *E. coli* and the expression levels were visualized by SDS-PAGE (Figure S5). The substitution of Y27 to the residue A, I, G, T, or S significantly caused the activity increase (>130%). Among them, the relative activity of the variant Y27S (designated as M_1_) was 1.6 times higher than that of the wild type (Figure 6a). The first round of iterative saturation mutation was conducted to determine whether the synergistic effect existed between the substitution Y27S and the residue V212. The wild type and the resulting variants were expressed in *E. coli*, and their crude enzyme activities were determined for evaluation (Figure S6). The activities of the variants Y27S/V212H, Y27S/V212S, Y27S/V212K and Y27S/V212R were greater than that of M_1_. Particularly, the double substitution of Y27S/V212R (designated as M_2_) caused an activity increase of 145% (Figure 6b). Similarly, the second round of iterative saturation mutation was conducted to determine the synergistic effect between the double substitution Y27S/V212R and the residue V111 (Figure S7). The variants Y27S/V212R/V111S, Y27S/V212R/V111I, Y27S/V212R/V111A and Y27S/V212R/V111G possessed higher activity than M_2_, and the triple substitution Y27S/V212R/V111G led to the best variant (Figure 6c).

**Figure 7:**
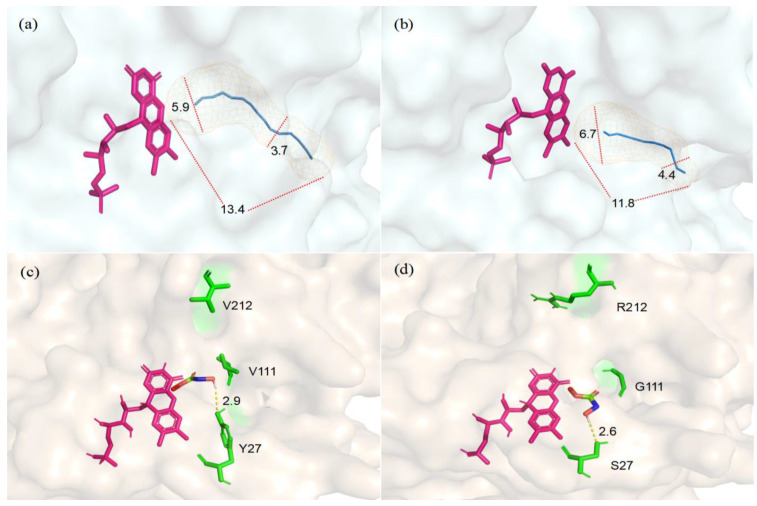
Structural comparison between the enzyme CreGO and its variant CreGO-Y27S/V212R/V11G through the docking analysis. (**a**) The substrate entry channel in CreGO; (**b**) the substrate entry channel in CreGO-Y27S/V212R/V11G; (**c**) the hydrogen bond between Y27 and the substrate in CreGO; (**d**) the hydrogen bond between S27 and the substrate in CreGO-Y27S/V212R/V11G.

To optimize the structural model of CreGO, the protein model was protonated and the energy was minimized using Schrödinger’s protein preparation function. The molecular docking analysis was conducted to explore the structural changes that originated from the triple substitution Y27S/V212R/V11G (Figure 7). Compared with the enzyme CreGO, the length of the substrate entry channel in the variant CreGO-Y27S/V212R/V11G was reduced from 13.4 Å to 11.8 Å. Furthermore, the maximum/minimum diameters of the channel were enlarged from 5.9 Å and 3.7 Å to 6.7 Å and 4.4 Å, respectively, implying that the structural changes benefited from the substrate entry to the active site. In addition, it was found that the substitution Y27S shortened the hydrogen bond between the hydroxyl group and the substrate from 2.9 Å to 2.6 Å, a change that might strengthen the enzyme-substrate interaction.

### 3.5. Purification and Characterization of GST-GSG-CreGO and Its Variant GST-GSG-CreGO-Y27S/V212R/V11G

**Figure 8:**
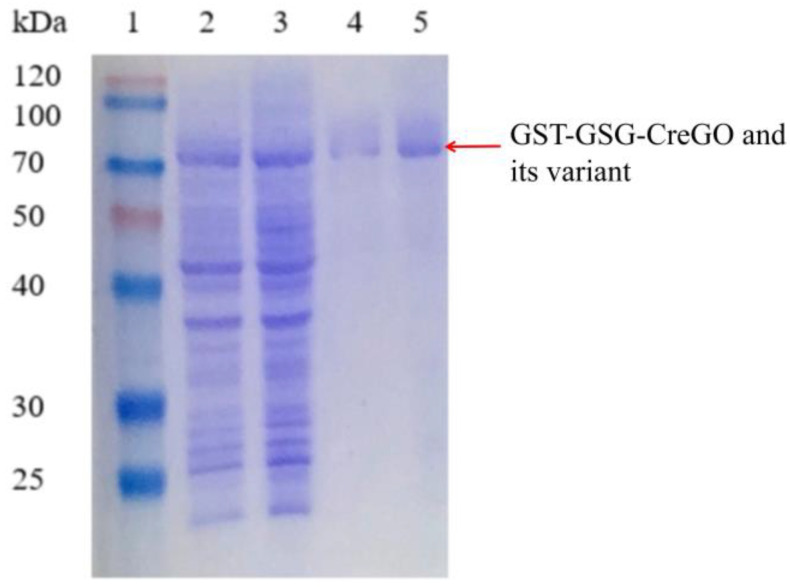
SDS-PAGE analysis of the purified GST-GSG-CreGO and its variant GST-GSG-CreGO-Y27S/V212R/V111G. Lane 1, standard molecular mass proteins; lane 2, crude GST-GSG-CreGO; lane 3, crude GST-GSG-CreGO-Y27S/V212R/V111G; lane 4, purified GST-GSG-CreGO; lane 5, purified GST-GSG-CreGO-Y27S/V212R/V111G. The proteins were visualized by staining with Coomassie brilliant blue R-250.

**Figure 9:**
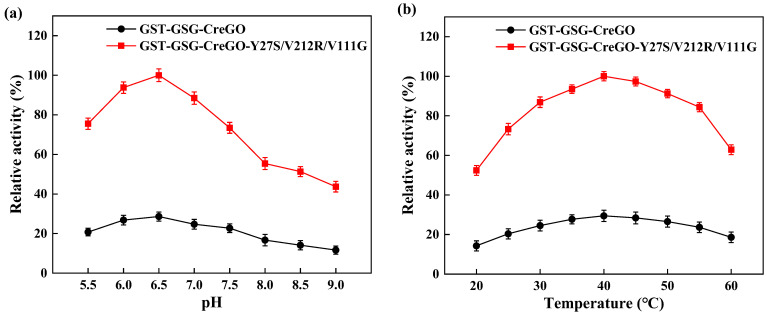
Effects of pH (**a**) and temperature (**b**) on the activities of purified GST-GSG-CreGO and GST-GSG-CreGO-Y27S/V212R/V111G. The relative activity of 100% represents 24.90 U/mg. Standard deviations are indicated in the diagram (*n* = 3).

Besides glycolate oxidases, glycolate dehydrogenases are capable of converting glycolate to glyoxylate in the presence of nicotinamide coenzyme [8,11,40,41]. To eliminate the effect of isoenzyme(s), both the enzymes GST-GSG-CreGO and GST-GSG-CreGO-Y27S/V212R/V11G were purified through affinity chromatography (Figure 8). The specific activities of purified GST-GSG-CreGO and GST-GSG-CreGO-Y27S/V212R/V11G were determined to be 6.9 U/mg and 24.9 U/mg, respectively. The effects of various factors (pH, temperature, metal ions and organic solvents) were investigated on the activity. The effect of the pH was determined within a range of 5.5~9.0 and the highest activity of both enzymes was 6.5 (Figure 9a). To determine the effect of temperature on the activity, the enzyme assay was conducted at temperatures ranging from 20 to 60 °C. The highest activity of both enzymes was observed at 40 °C (Figure 9b). The effects of metal chelating agent EDTA and metal ions (Na^+^, Zn^2+^, Mg^2+^, Mn^2+^, K^+^ and Ca^2+^) were explored. Among them, Mg^2+^ and Mn^2+^ promoted the activity of both enzymes. The effects of EDTA and Na^+^ were not significant, whereas Ca^2+^, Zn^2+^ and K^+^ inhibited the activity (Figure S8). Using the sample without an organic solvent as the control, the effects of organic reagents (methanol, ethanol, isopropyl alcohol, n-propanol, polyethylene glycol PEG-400 and DMSO) on the activity were investigated. Both enzymes were inhibited by the tested organic solvents to some extent (Figure S9). Obeying the Michaelis–Menton kinetics, the kinetic parameters of both enzymes were determined within the substrate range of 0~100 mM (Table 1). The *K*_m_ values of GST-GSG-CreGO and GST-GSG-CreGO-Y27S/V212R/V11G were 1.0 mM and 0.6 mM, suggesting that the triple substitution led to the superior affinity to the substrate. Moreover, the *k*_cat_/*K*_m_ value of GST-GSG-CreGO-Y27S/V212R/V11G (29.2 s^−1^ mM^−1^) was six times higher than that of GST-GSG-CreGO (4.9 s^−1^ mM^−1^). Under the optimized conditions, the induction of the strain *E. coli* BL21(DE3)/pET22b-GST-GSG-CreGO-Y27S/V212R/V11G led to the crude enzyme activity of 0.49 U/mg, proving that the semi-rational design benefited the enhancement of both the catalytic activity and enzyme productivity.

### 3.6. Oxidation of Methyl Glycolate Catalyzed by Crude Enzymes GST-GSG-CreGO and GST-GSG-CreGO-Y27S/V212R/V111G

**Figure 10:**
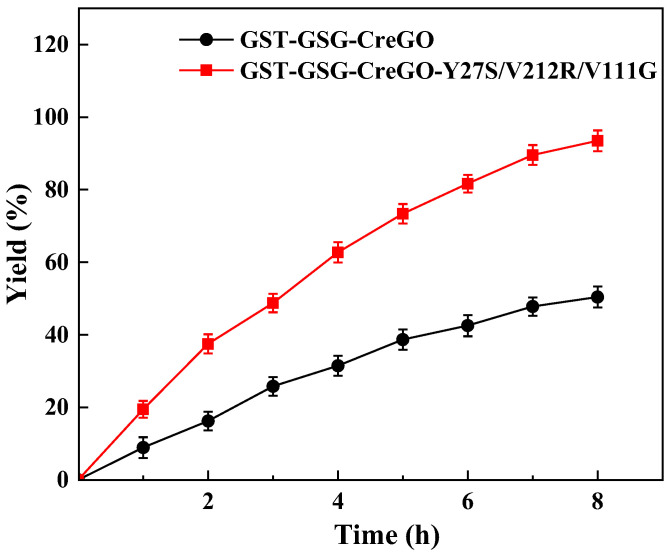
Oxidation of 300 mM methyl glycolate catalyzed by crude enzymes GST-GSG-CreGO and GST-GSG-CreGO-Y27S/V212R/V111G. Standard deviations are indicated in the diagram (*n* = 3).

Catalytic performances of GST-GSG-CreGO and GST-GSG-CreGO-Y27S/V212R/V111G were evaluated by the oxidation of methyl glycolate rather than that of sodium glycolate in enzyme assay. The biocatalyst was used in the form of a crude enzyme. The use of a crude enzyme virtually eliminated side reactions observed in whole-cell catalysis, and its preparation was much simpler than that of purified enzymes [8]. The oxidation of methyl glycolate led to the byproduct H_2_O_2_, which is detrimental to the activity [28]. To decompose H_2_O_2_, the *E. coli* strain expressing the catalase from *Acinetobacter* sp. YS0810 was constructed and induced (Figure S10), and then the crude catalase was prepared. The specific activity of the crude catalase was as high as 527.1 U/mg. In addition, the supply of oxygen is crucial for the catalytic efficiency of the biocatalytic oxidation reaction [3]. Considering the aforementioned factors, the optimized reaction mixture consisted of 5 mg/mL crude glycolate oxidase (GST-GSG-CreGO or GST-GSG-CreGO-Y27S/V212R/V111G), 25 μg/mL crude catalase, 300 mM methyl glycolate and 100 mM PBS buffer (pH 6.5). The reaction was run at 40 °C and 1000 rpm for 8 h, maintaining an oxygenation rate of 1.4 L/h (Figure 10). When the reaction was terminated, the yields for GST-GSG-CreGO and GST-GSG-CreGO-Y27S/V212R/V111G catalyzed reactions were 50.4% and 93.5%, respectively, indicating that the semi-rational design was effective for the enhancement of catalytic activity.

## 4. Conclusions

The uncharacterized glycolate oxidase from *Chlamydomonas reinhardtii* was selected through phylogenetic analysis, and its expression in *E. coli* led to a crude enzyme activity of 0.06 U/mg. The implementation of combined strategies, including the fusion of the GST tag, the choice of vector and the optimization of induction conditions, increased the crude enzyme activity by up to 0.15 U/mg. Using the fusion enzyme GST-GSG-CreGO as a template, the triple substitution Y27S/V212R/V111G was introduced to improve the catalytic activity through a semi-rational design. The fusion enzyme GST-GSG-CreGO and its variant GST-GSG-CreGO-Y27S/V212R/V111G were purified and then investigated for data. The *k*_cat_/*K*_m_ value of GST-GSG-CreGO-Y27S/V212R/V11G was calculated to be 29.2 s^−1^ mM^−1^, six times higher than that of GST-GSG-CreGO (4.9 s^−1^ mM^−1^). The docking analysis suggested that the enhancement of catalytic efficiency might be attributed to the enlargement of the substrate entry channel and the strengthened interaction with the substrate. Finally, the crude enzymes GST-GSG-CreGO and GST-GSG-CreGO-Y27S/V212R/V111G catalyzed the oxidation of 300 mM methyl glycolate to methyl glyoxylate and afforded yields of 50.4% and 93.5%, respectively. The construction of the strain *E. coli* BL21(DE3)/pET22b-GST-GSG-CreGO-Y27S/V212R/V11G increased the productivity of crude enzyme up to 0.49 U/mg, demonstrating that the semi-rational design of GST-GSG-CreGO benefited the enhancement of both catalytic activity and enzyme productivity.

## Figures and Tables

**Table 1:** The kinetic parameters of purified glycolate oxidases GST-GSG-CreGO and GST-GSG-CreGO-Y27S/V212R/V111G ^a^.

Parameters	GST-GSG-CreGO	GST-GSG-CreGO-Y27S/V212R/V111G
*V*_max_ (U/mg)	7.2 ± 0.2	25.5 ± 0.9
*K*_m_ (mM)	1.0 ± 0.05	0.6 ± 0.04
*k*_cat_ (s^−1^)	4.9 ± 0.2	17.5 ± 0.6
*k*_cat_/*K*_m_ (s^−1^·mM^−1^)	4.9 ± 0.2	29.2 ± 1.0

^a^ Standard deviations are indicated in the table (*n* = 3).

## Data Availability

The data presented in this study are available in Supplementary Materials.

## Supplementary Materials

The following supporting information can be downloaded at: https://www.mdpi.com/article/10.3390/microorganisms11071689/s1, Table S1: The primers for the fusion expression of CreGO and GST; Table S2: The primers for site-directed mutagenesis of CreGO in alanine scanning; Table S3: The primers for site-directed saturation mutagenesis of Y27 in CreGO; Table S4: The primers for iterative saturation mutagenesis of V212 in CreGO; Table S5: The primers for iterative site-saturation mutagenesis of V111 in CreGO; Figure S1: HPLC analyses of methyl glycolate (a) and methyl glyoxylate (b); Figure S2: Modeling of glycolate oxidase CreGO; Figure S3: Ramachandran plot analysis of CreGO modeling; Figure S4: SDS-PAGE (12%) analysis of the fusion enzyme GST-GSGCreGO and its variants in alanine scanning; Figure S5: SDS-PAGE (12%) analysis of the fusion enzyme GST-GSG-CreGO and its variants in the saturation mutagenesis of the residue Y27; Figure S6: SDS-PAGE (12%) analysis of the glycolate oxidase GST-GSG-CreGO-Y27S and its variants in the iterative saturation mutagenesis of the residue V212; Figure S7: SDS-PAGE (12%) analysis of the glycolate oxidase GST-GSG-CreGO-Y27S/V212R and its variants in the iterative saturation mutagenesis of the residue V111; Figure S8: The effects of metal ions and EDTA on activity of GST-GSGCreGO and its variant GST-GSG-CreGO-Y27S/V212R/V111G; Figure S9: The effects of organic solvents on activity of GST-GSG-CreGO and its variant GST-GSG-CreGO-Y27S/V212R/V111G; Figure S10: SDS-PAGE (12%) analysis of recombinant catalase from Acinetobacter sp. YS0810 expressed in *E. coli*.
