# Supplementary material for: Enhancing the Catalytic Activity of Glycolate Oxidase from Chlamydomonas reinhardtii through Semi-Rational Design
Source: Microorganisms. 2023 Jun 28;11(7):1689. doi: 10.3390/microorganisms11071689 (PMC10385363; doi:10.3390/microorganisms11071689)
Supplement: Supplementary file 1 [file microorganisms-11-01689-s001.zip › microorganisms-2437046-supplementary.pdf]

Supporting Information

Enhancing the Catalytic Activity of of Glycolate Oxidase from *Chlamydomonas reinhardtii* through Semi-rational Design

Yingting Feng [1,†], Shuai Shao [1,†], Xueting Zhou [1], Wan Wei [1], Xun Liu [1], Yi Tang [1], Yuhao Hua [1], Jianyong Zheng [1], Yinjun Zhang [1,*] and Xiangxian Ying [1,*]

1 Key Laboratory of Bioorganic Synthesis of Zhejiang Province, College of Biotechnology and Bioengineering, Zhejiang University of Technology, Hangzhou 310014, China; fyt9191@163.com (Y.F.); ss1964241742@163.com (S.S.); sadie598851321@163.com (X.Z.); zjut2112105022@163.com (W.W.); lx0925124X@163.com (X.L.); ty12150102@163.com (Y.T.); hyhua0616@163.com (Y.H.); zjy821212@zjut.edu.cn (J.Z.)

* Correspondence: jsun@zjut.edu.cn (J.S.); yingxx@zjut.edu.cn (X.Y.)

† These authors contributed equally to this work.

Table of Contents

Supplementary tables 2

Table S1 The primers for the fusion expression of CreGO and GST 2

Table S2 The primers for site-directed mutagenesis of CreGO in alanine scanning 3

Table S3 The primers for site-directed saturation mutagenesis of Y27 in CreGO 4

Table S4 The primers for iterative saturation mutagenesis of V212 in CreGO 6

Table S5 The primers for iterative site-saturation mutagenesis of V111 in CreGO 8

Supplementary figures 10

Figure S1 HPLC analyses of methyl glycolate (a) and methyl glyoxylate (b). 10

Figure S2 Modeling of glycolate oxidase CreGO 11

Figure S3 Ramachandran plot analysis of CreGO modeling 12

Figure S4 SDS-PAGE (12%) analysis of the fusion enzyme GST-GSG-CreGO and its variants in alanine scanning 13

Figure S5 SDS-PAGE (12%) analysis of the fusion enzyme GST-GSG-CreGO and its variants in the saturation mutagenesis of the residue Y27. 14

Figure S6 SDS-PAGE (12%) analysis of the glycolate oxidase GST-GSG-CreGO-Y27S and its variants in the iterative saturation mutagenesis of the residue V212 15

Figure S7 SDS-PAGE (12%) analysis of the glycolate oxidase GST-GSG-CreGO-Y27S/V212R and its variants in the iterative saturation mutagenesis of the residue V111 16

Figure S8 The effects of metal ions and EDTA on activity of GST-GSG-CreGO and its variant GST-GSG-CreGO-Y27S/V212R/V111G 17

Figure S9 The effects of organic solvents on activity of GST-GSG-CreGO and its variant GST-GSG-CreGO-Y27S/V212R/V111G 18

Figure S10 SDS-PAGE (12%) analysis of recombinant catalase from *Acinetobacter* sp. YS0810 expressed in *E. coli* 19

Supplementary tables

Table S1 The primers for the fusion expression of CreGO and GST

Primer	Sequence (5’-3’)
pET22b-GST-F	AAGCTTGCGGCCGCACTC
pET22b-GST-GSG-R	TCCACTTCCACGCGGAACCAGATCGCTTT
GSG-CreGO-F	AAAAGCGATCTGGTTCCGCGTGGAAGTGGAATGGCAGACCTGAGCTTTCTGA
CreGO-R	CTCGAGTGCGGCCGCAAGCTTCAGTTTACACAGCTGTGCTGCC

Table S2 The primers for site-directed mutagenesis of CreGO in alanine scanning

Primer	Sequence (5′-3′)
Y27A-F	TTTGATGCATATAGCACCGGTAGCGATACCTGT
Y27A-R	GCTATATGCATCAAATGCCATTTTCGGCATA
V111A-F	AGCACCGCAGCAACCAGCAGCCTGCAGG
V111A-R	GGTTGCTGCGGTGCTAAAGGTAAACGGAACACCT
L164A-F	CAGCGTGCAGGTAATCGTGAAGCAGATGCACG
L164A-R	ACGATTTGCACCACGCTGTGCATCAACGGTAACC
R167 A-F	GGTAATGCAGAAGCAGATGCACGTAATAAATTTACC
R167 A-R	TGCTTTGCGATTACCCAGACGCTGTGCATCAAC
F208A-F	AAACTGGCAACCAGCGAAGTTGATGATAGCCTG
F208A-R	GCTGGTTGCCAGTTTCATCAGACCGCTACCATCCTG
V212A-F	AGCGAAGCAGATGATAGCCTGACCTGGGAATTTATT
V212A-R	ATCATCTGCTTCGCTGGTAAACAGTTTCATCAGAC

Table S3 The primers for site-directed saturation mutagenesis of Y27 in CreGO

Primer	Sequence (5'-3')
Y27S-F	TTTGATAGCTATAGCACCGGTAGCGATACCTGT
Y27S-R	GCTATAGCTATCAAATGCCATTTTCGGCATA
Y27T-F	TTTGATACCTATAGCACCGGTAGCGATACCTGT
Y27T-R	GCTATAGGTATCAAATGCCATTTTCGGCATA
Y27C-F	TTTGATTGCTATAGCACCGGTAGCGATACCTGT
Y27C-R	GCTATAGCAATCAAATGCCATTTTCGGCATA
Y27N-F	TTTGATAATTATAGCACCGGTAGCGATACCTGT
Y27N-R	GCTATAATTATCAAATGCCATTTTCGGCATA
Y27Q-F	TTTGATCAGTATAGCACCGGTAGCGATACCTGT
Y27Q-R	GCTATA CTGATCAAATGCCATTTTCGGCATA
Y27H-F	TTTGATCATTATAGCACCGGTAGCGATACCTGT
Y27H-R	GCTATAATGATCAAATGCCATTTTCGGCATA
Y27K-F	TTTGATAAATATAGCACCGGTAGCGATACCTGT
Y27K-R	GCTATATTTATCAAATGCCATTTTCGGCATA
Y27R-F	TTTGATCGTTATAGCACCGGTAGCGATACCTGT
Y27R-R	GCTATAACGATCAAATGCCATTTTCGGCATA
Y27D-F	TTTGATGATTATAGCACCGGTAGCGATACCTGT
Y27D-R	GCTATAATCATCAAATGCCATTTTCGGCATA
Y27E-F	TTTGATGAATATAGCACCGGTAGCGATACCTGT
Y27E-R	GCTATATTCATCAAATGCCATTTTCGGCATA
Y27V-F	TTTGATGTTTATAGCACCGGTAGCGATACCTGT
Y27V-R	GCTATAAACATCAAATGCCATTTTCGGCATA
Y27L-F	TTTGATCTGTATAGCACCGGTAGCGATACCTGT
Y27L-R	GCTATACAGATCAAATGCCATTTTCGGCATA
Y27I-F	TTTGATATTTATAGCACCGGTAGCGATACCTGT
Y27I-R	GCTATAAATATCAAATGCCATTTTCGGCATA
Y27F-F	TTTGATTTTTATAGCACCGGTAGCGATACCTGT
Y27F-R	GCTATAAAAATCAAATGCCATTTTCGGCATA

Y27W-F	TTTGATTGGTATAGCACCGGTAGCGATACCTGT
Y27W-R	GCTATACCAATCAAATGCCATTTTCGGCATA
Y27M-F	TTTGATATGTATAGCACCGGTAGCGATACCTGT
Y27M-R	GCTATACATATCAAATGCCATTTTCGGCATA
Y27P-F	TTTGATCCGTATAGCACCGGTAGCGATACCTGT
Y27P-R	GCTATACGGATCAAATGCCATTTTCGGCATA

Table S4 The primers for iterative saturation mutagenesis of V212 in CreGO

Primer	Sequence (5'-3')
V212S-F	AGCGAAAGCGATGATAGCCTGACCTGGGAATTTATT
V212S-R	ATCATCGCTTTCGCTGGTAAACAGTTTCATCAGAC
V212T-F	AGCGAAACCGATGATAGCCTGACCTGGGAATTTATT
V212T-R	ATCATCGGTTTCGCTGGTAAACAGTTTCATCAGAC
V212C-F	AGCGAATGCGATGATAGCCTGACCTGGGAATTTATT
V212C-R	ATCATCGCATTCGCTGGTAAACAGTTTCATCAGAC
V212N-F	AGCGAAAATGATGATAGCCTGACCTGGGAATTTATT
V212N-R	ATCATCATTTTCGCTGGTAAACAGTTTCATCAGAC
V212Q-F	AGCGAACAGGATGATAGCCTGACCTGGGAATTTATT
V212Q-R	ATCATCCTGTTCGCTGGTAAACAGTTTCATCAGAC
V212H-F	AGCGAACATGATGATAGCCTGACCTGGGAATTTATT
V212H-R	ATCATCATGTTCGCTGGTAAACAGTTTCATCAGAC
V212K-F	AGCGAAAAAGATGATAGCCTGACCTGGGAATTTATT
V212K-R	ATCATCTTTTTCGCTGGTAAACAGTTTCATCAGAC
V212R-F	AGCGAACGTGATGATAGCCTGACCTGGGAATTTATT
V212R-R	ATCATCACGTTCGCTGGTAAACAGTTTCATCAGAC
V212D-F	AGCGAAGATGATGATAGCCTGACCTGGGAATTTATT
V212D-R	ATCATCATCTTCGCTGGTAAACAGTTTCATCAGAC
V212E-F	AGCGAAGAAGATGATAGCCTGACCTGGGAATTTATT
V212E-R	ATCATCTTCTTCGCTGGTAAACAGTTTCATCAGAC
V212L-F	AGCGAACTGGATGATAGCCTGACCTGGGAATTTATT
V212L-R	ATCATCCAGTTCGCTGGTAAACAGTTTCATCAGAC
V212I-F	AGCGAAATTGATGATAGCCTGACCTGGGAATTTATT
V212I-R	ATCATCAATTTCGCTGGTAAACAGTTTCATCAGAC
V212F-F	AGCGAATTTGATGATAGCCTGACCTGGGAATTTATT
V212F-R	ATCATCAAATTCGCTGGTAAACAGTTTCATCAGAC
V212W-F	AGCGAATGGGATGATAGCCTGACCTGGGAATTTATT
V212W-R	ATCATCCCATTCGCTGGTAAACAGTTTCATCAGAC

V212M-F	AGCGAAATGGATGATAGCCTGACCTGGGAATTTATT
V212M-R	ATCATCCATTTCGCTGGTAAACAGTTTCATCAGAC
V212P-F	AGCGAACCGGATGATAGCCTGACCTGGGAATTTATT
V212P-R	ATCATCCGGTTCGCTGGTAAACAGTTTCATCAGAC
V212Y-F	AGCGAATATGATGATAGCCTGACCTGGGAATTTATT
V212Y-R	ATCATCATATTCGCTGGTAAACAGTTTCATCAGAC
V212A-F	AGCGAAGCAGATGATAGCCTGACCTGGGAATTTATT
V212A-R	ATCATCTGCTTCGCTGGTAAACAGTTTCATCAGAC

Table S5 The primers for iterative site-saturation mutagenesis of V111 in CreGO

Primer	Sequence (5'-3')
V111S-F	AGCACCAGCGCAACCAGCAGCCTGCAGG
V111S-R	GGTTGCGCTGGTGCTAAAGGTAAACGGAACACCT
V111T-F	AGCACCACCGCAACCAGCAGCCTGCAGG
V111T-R	GGTTGCGGTGGTGCTAAAGGTAAACGGAACACCT
V111C-F	AGCACCTGCGCAACCAGCAGCCTGCAGG
V111C-R	GGTTGCGCAGGTGCTAAAGGTAAACGGAACACCT
V111N-F	TTTGATAATTATAGCACCGGTAGCGATACCTGT
V111N-R	GGTTGCATTGGTGCTAAAGGTAAACGGAACACCT
V111Q-F	AGCACCCAGGCAACCAGCAGCCTGCAGG
V111Q-R	GGTTGCCTGGGTGCTAAAGGTAAACGGAACACCT
V111H-F	AGCACCCATGCAACCAGCAGCCTGCAGG
V111H-R	GGTTGCATGGGTGCTAAAGGTAAACGGAACACCT
V111K-F	AGCACCAAAGCAACCAGCAGCCTGCAGG
V111K-R	GGTTGCTTTGGTGCTAAAGGTAAACGGAACACCT
V111R-F	TTTGATCGTTATAGCACCGGTAGCGATACCTGT
V111R-R	GGTTGCACGGGTGCTAAAGGTAAACGGAACACCT
V111D-F	TTTGATGATTATAGCACCGGTAGCGATACCTGT
V111D-R	GGTTGCATCGGTGCTAAAGGTAAACGGAACACCT
V111E-F	AGCACCGAAGCAACCAGCAGCCTGCAGG
V111E-R	GGTTGCTTCGGTGCTAAAGGTAAACGGAACACCT
V111V-F	TTTGATGTTTATAGCACCGGTAGCGATACCTGT
V111V-R	AGCACCCTGGCAACCAGCAGCCTGCAGG
V111L-F	TTTGATCTGTATAGCACCGGTAGCGATACCTGT
V111L-R	GGTTGCCAGGGTGCTAAAGGTAAACGGAACACCT
V111I-F	AGCACCATTGCAACCAGCAGCCTGCAGG
V111I-R	GGTTGCAATGGTGCTAAAGGTAAACGGAACACCT
V111F-F	AGCACCTTTGCAACCAGCAGCCTGCAGG
V111F-R	GGTTGCAAAGGTGCTAAAGGTAAACGGAACACCT

V111W-F	AGCACCTGGGCAACCAGCAGCCTGCAGG
V111W-R	GGTTGCCCAGGTGCTAAAGGTAAACGGAACACCT
V111M-F	AGCACCATGGCAACCAGCAGCCTGCAGG
V111M-R	GGTTGCCATGGTGCTAAAGGTAAACGGAACACCT
V111P-F	AGCACCCCGGCAACCAGCAGCCTGCAGG
V111P-R	GGTTGCCGGGGTGCTAAAGGTAAACGGAACACCT
V111Y-F	AGCACCTATGCAACCAGCAGCCTGCAGG
V111Y-R	GGTTGCATAGGTGCTAAAGGTAAACGGAACACCT
V111A-F	AGCACCGCAGCAACCAGCAGCCTGCAGG
V111A-R	GGTTGCTGCGGTGCTAAAGGTAAACGGAACACCT

Supplementary figures

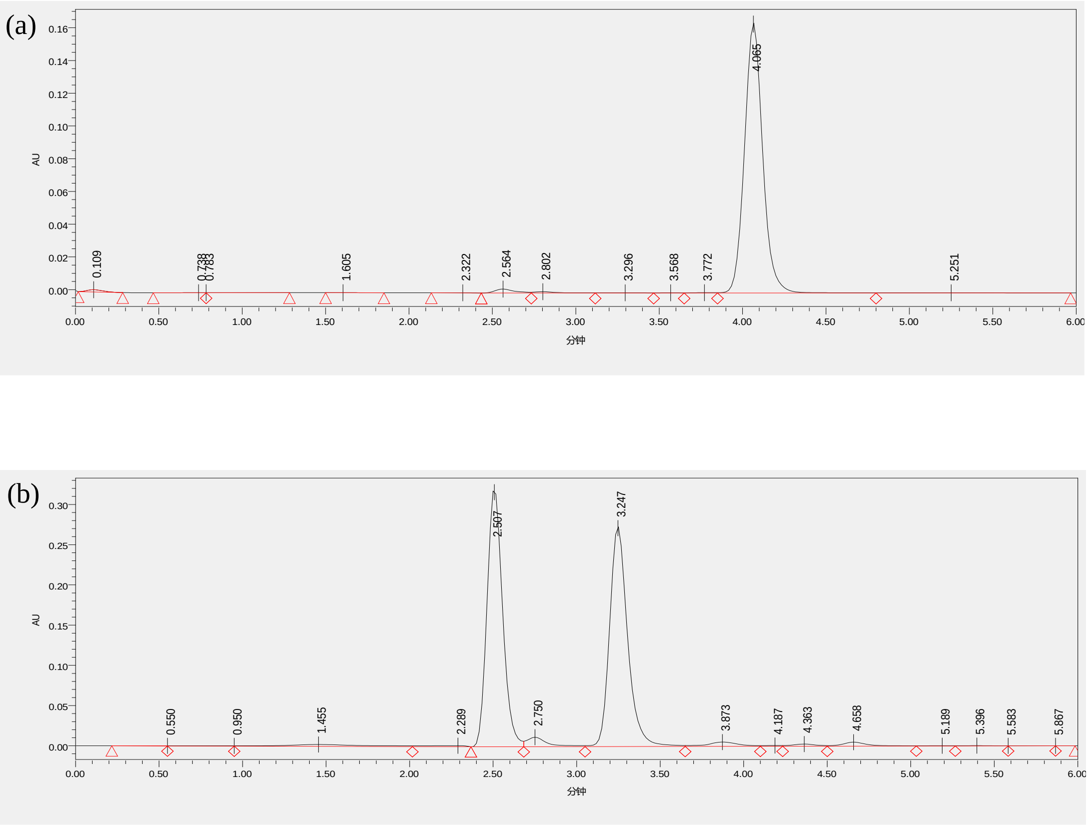

Figure S1 HPLC analyses of methyl glycolate (a) and methyl glyoxylate (b). Methyl glycolate, 4.07 min; methyl glyoxylate, 2.51 min; toluene as solvent, 3.25 min.

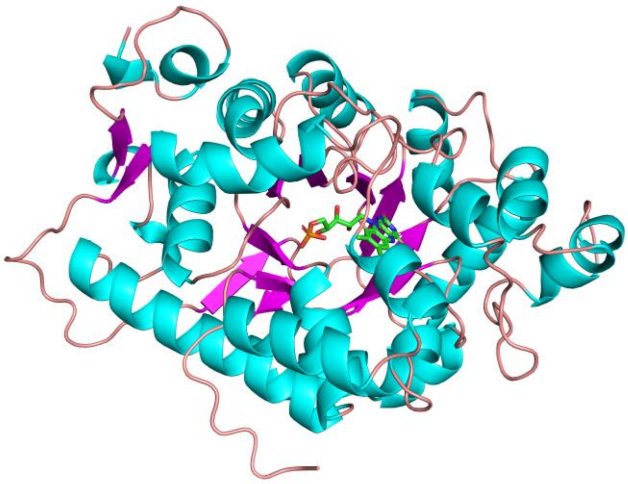

Figure S2 Modeling of glycolate oxidase CreGO

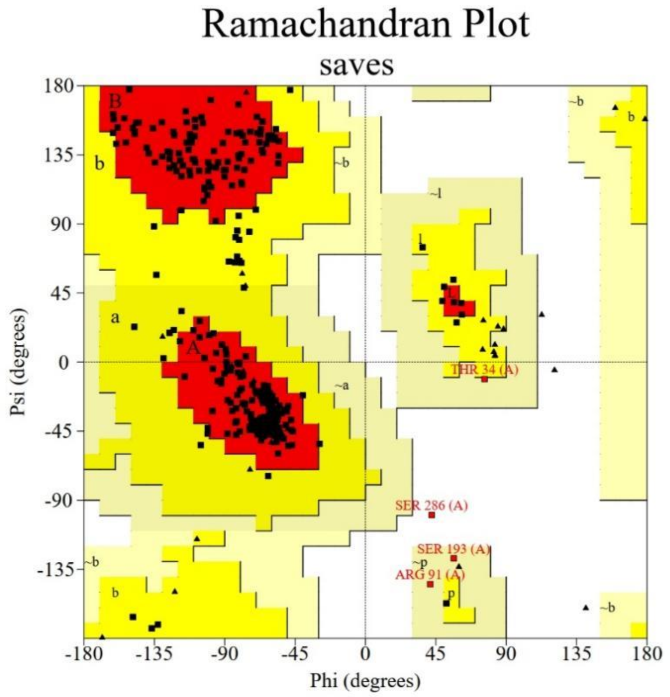

Plot statistics

Residues in most favoured regions [A,B,L]	286	88.3%
Residues in additional allowed regions [a,b,l,p]	34	10.5%
Residues in generously allowed regions [~a,~b,~l,~p]	3	0.9%
Residues in disallowed regions	1	0.3%
	----	------
Number of non-glycine and non-proline residues	324	100.0%
Number of end-residues (excl. Gly and Pro)	4	
Number of glycine residues (shown as triangles)	34	
Number of proline residues	22	

Total number of residues	384	

Figure S3 Ramachandran plot analysis of CreGO modeling

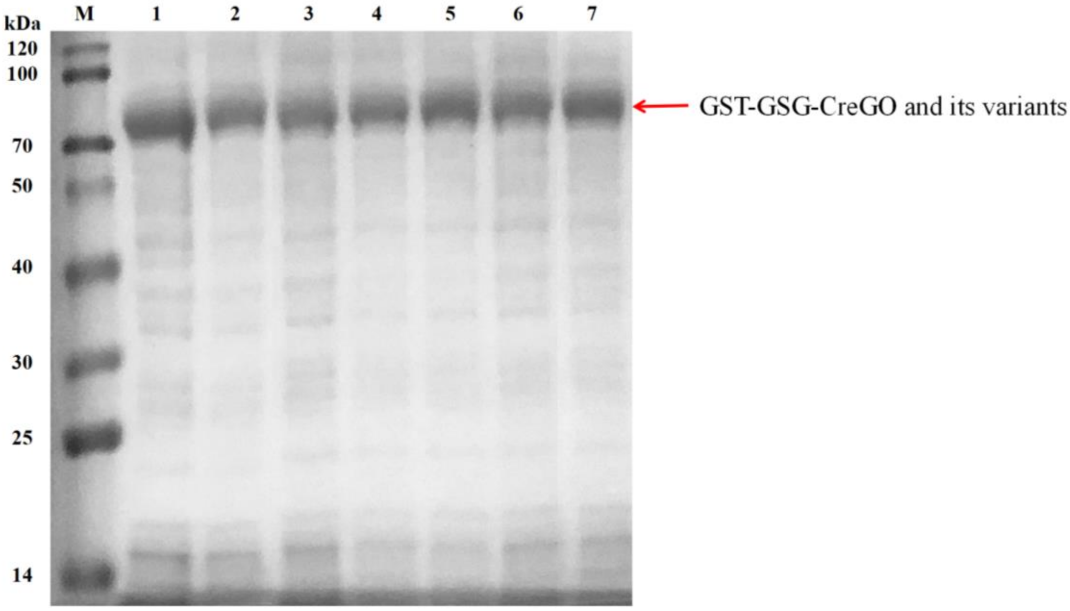

Figure S4 SDS-PAGE (12%) analysis of the fusion enzyme GST-GSG-CreGO and its variants in alanine scanning. Lane M, standard molecular mass proteins; lanes from 1 to 7 represent the variants (from left to right): GST-GSG-CreGO, Y27A, V111A, L164A, R167A, F208A and V212A. The proteins were visualized by staining with Coomassie brilliant blue R-250.

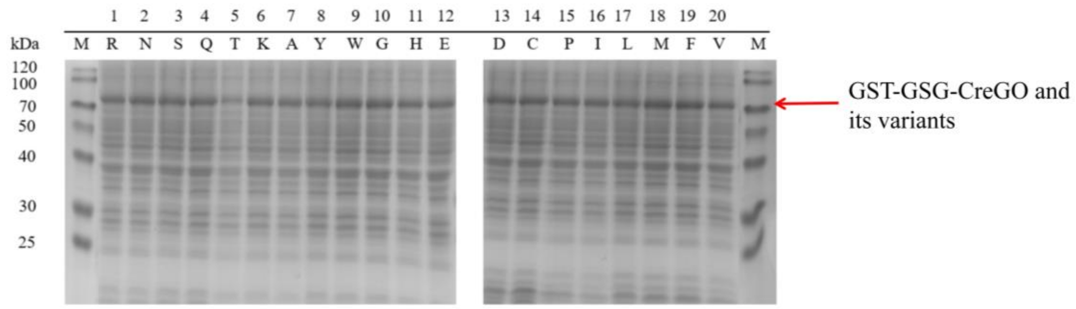

Figure S5 SDS-PAGE (12%) analysis of the fusion enzyme GST-GSG-CreGO and its variants in the saturation mutagenesis of the residue Y27. Lane M, standard molecular mass proteins; lanes from 1 to 20 represent the variants (from left to right): Y27R, Y27N, Y27S, Y27Q, Y27T, Y27K, Y27A, wild type, Y27W, Y27G, Y27H, Y27E, Y27D, Y27C, Y27P, Y27I, Y27L, Y27M, Y27F and Y27V. The proteins were visualized by staining with Coomassie brilliant blue R-250.

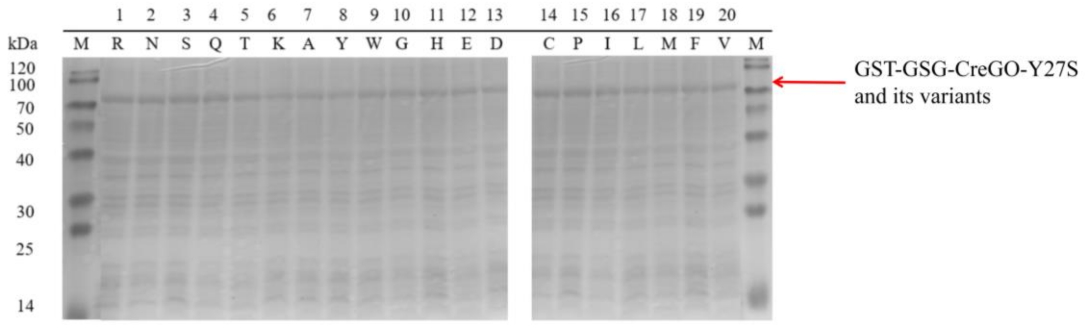

Figure S6 SDS-PAGE (12%) analysis of the glycolate oxidase GST-GSG-CreGO-Y27S and its variants in the iterative saturation mutagenesis of the residue V212. Lane M, standard molecular mass proteins; lanes from 1 to 20 represent the variants (from left to right): V212R, V212N, V212S, V212Q, V212T, V212K, V212A, V212Y, V212W, V212G, V212H, V212E, V212D, V212C, V212P, V212I, V212L, V212M, V212F and M_1. The proteins were visualized by staining with Coomassie brilliant blue R-250.

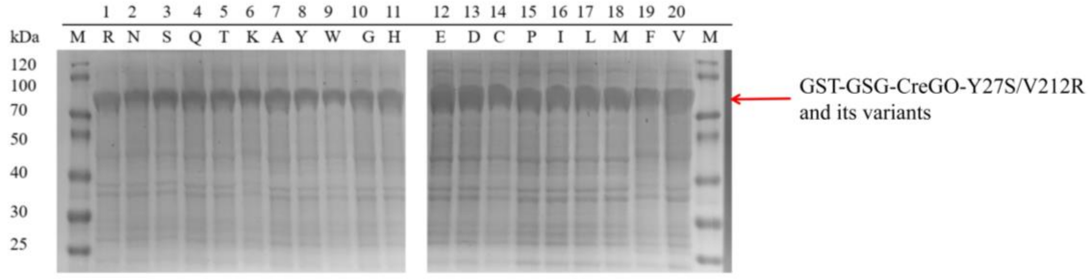

Figure S7 SDS-PAGE (12%) analysis of the glycolate oxidase GST-GSG-CreGO-Y27S/V212R and its variants in the iterative saturation mutagenesis of the residue V111. Lane M, standard molecular mass proteins; lanes from 1 to 20 represent the variants (from left to right): V111R, V111N, V111S, V111Q, V111T, V111K, V111A, V111Y, V111W, V111G, V111H, V111E, V111D, V111C, V111P, V111I, V111L, V111M, V111F and M_2. The proteins were visualized by staining with Coomassie brilliant blue R-250.

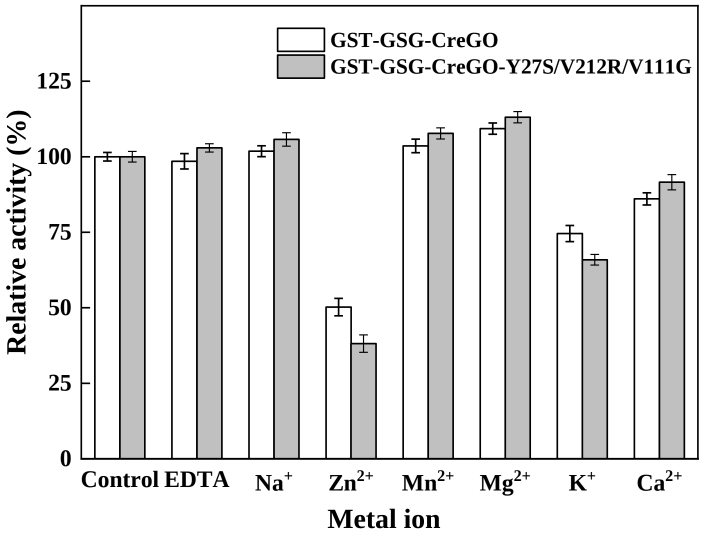

Figure S8 The effects of metal ions and EDTA on activity of GST-GSG-CreGO and its variant GST-GSG-CreGO-Y27S/V212R/V111G. The assay mixture (1 mL) consisted of 1 μg purified enzyme, 10 mM phenylhydrazine hydrochloride, 1 mM metal ion or EDTA, 10 mM sodium glycolate and 100 mM PBS buffer (pH 6.5). The activity was measured in triplicate at 40 °C by monitoring changes in the absorbance at 324 nm. The relative activity of 100% for GST-GSG-CreGO and GST-GSG-CreGO-Y27S/V212R/V111G represents 6.9 U/mg and 24.9 U/mg, respectively. Standard deviations are indicated in the diagram (n = 3).

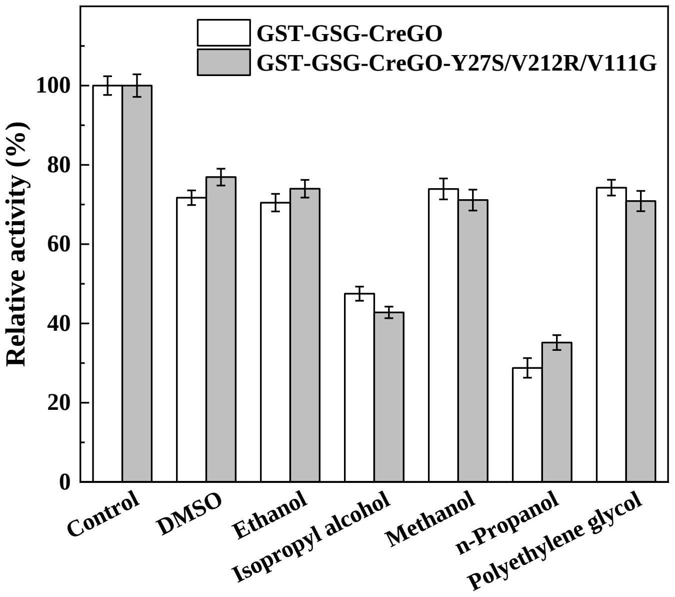

Figure S9 The effects of organic solvents on activity of GST-GSG-CreGO and its variant GST-GSG-CreGO-Y27S/V212R/V111G. The assay mixture (1 mL) consisted of 1 μg purified enzyme, 10 mM phenylhydrazine hydrochloride, 10% (v/v) organic solvents, 10 mM sodium glycolate and 100 mM PBS buffer (pH 6.5). The activity was measured in triplicate at 40 °C by monitoring changes in the absorbance at 324 nm. The relative activity of 100% for GST-GSG-CreGO and GST-GSG-CreGO-Y27S/V212R/V111G represents 6.9 U/mg and 24.9 U/mg. Standard deviations are indicated in the diagram (*n* = 3).

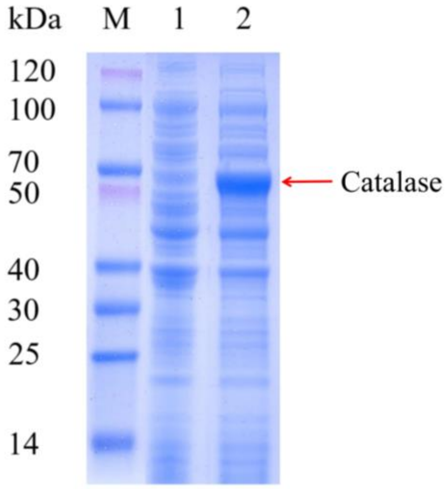

Figure S10 SDS-PAGE (12%) analysis of recombinant catalase from *Acinetobacter* sp. YS0810 expressed in *E. coli*. The proteins were visualized by staining with Coomassie brilliant blue R-250.
